# Violent and Non-Violent Criminal Behavior among Young Chinese Drug Users: A Mixed Methods Study

**DOI:** 10.3390/ijerph15030432

**Published:** 2018-03-02

**Authors:** Liu Liu, Wing Hong Chui, Ye Chen

**Affiliations:** 1 School of Social and Behavioral Sciences, Nanjing University, No. 163, Xianlin Avenue, Qixia District, Nanjing 210023, China; chenye_199308@163.com; 2 Department of Applied Social Sciences, City University of Hong Kong, Tat Chee Avenue, Kowloon, Hong Kong, China; eric.chui@cityu.edu.hk

**Keywords:** young Chinese drug users, mixed methods, criminal experience, violence, mental health

## Abstract

Young drug users are found to be increasingly involved in criminal justice issues. This exploratory and descriptive study aims to analyze the criminal behaviors among young Chinese drug users through a mixed methods research design. Quantitative analysis indicates that young drug users with and without a history of criminality show significant differences in terms of several features. Male drug users, particularly, those who are older, with religious beliefs, and initiated into drug use at younger age were most likely to commit crimes. Among drug users with criminal experiences, those who committed crimes prior to drug initiation have a greater likelihood of committing violent crimes. Furthermore, young drug users with severe depression are more likely to commit crimes, especially violent ones. Qualitative analysis further illustrates that young male drug users often get involved in criminal conduct of the youth gang nature with propensity for engaging in violent crimes as compared to their female counterparts who are more likely to turn into drug dealers and traffickers, in addition to engaging in larceny. The research findings are consistent with developmental theories and “victim to offender cycle”. Integrated mental health and substance use services are suggested for crime prevention among young Chinese drug users.

## 1. Introduction

Research has established that drug use among young people has risen considerably in recent years [1,2,3,4], and China is no exception. Along with economic prosperity, Chinese society faces a rapidly growing population of drug users. Currently, drug use is regarded as one of the most pressing social problems in the country [5,6,7]. The registered drug users (individuals who have received, at least, one police citation for drug use) has grown substantially in a short period; from 2.1 million in 2012 [8] to 2.5 million by the end of 2016 [9]. Nearly 60% of the above-mentioned registered drug users were aged 35 or under in 2016 [9]. The statistics indicate not only that the number of drug users has surged dramatically in China, but also that young people represent a large proportion of the drug user population.

Substance abuse, which includes the use of drugs and alcohol, is a major public health problem around the globe [10]. According to the statistics of the World Health Organization (WHO), drug and alcohol use has an influence on 4% of deaths and accounts for 5.4% of the global burden of disease [11,12]. Research shows that long-term drug use can lead to serious health problems, especially significantly higher probability of mental disorders. Individuals who use heroin for long periods usually have poor mental and physical health [13], which manifest in such symptoms as anxiety, hepatitis, and tuberculosis [14]. Long-term methamphetamine use may result in dopamine reduction, which is associated with psychological disorders including depression, paranoia, psychosis, and mood problems [15,16]. 

The link between drug use and criminal behavior has also received continuous attention from scholars and policymakers, producing a large volume of literature [17,18,19]. There is a general perception of a close relationship between violence and drug consumption [20,21]. For example, prior studies have indicated that the use of methamphetamine, a non-opiate synthetic drug, may increase the likelihood of its users engaging in criminal behaviors, including assault, kidnapping, reckless driving, robbery, and homicide [22,23]. Empirical data suggest a strong association between young people’s drug use and criminality, even when dealing in drug and trafficking are disregarded [24,25,26]. On the whole, young drug users have been found to be increasingly involved in criminal justice-related issues [27,28]. The high rate of young drug users participating in violent and non-violent criminal activities is particularly worrying, because such behaviors are also often accompanied by a lower life expectancy as well as a difficult transition into normal adulthood [29,30].

The rapid rise in the number of young Chinese drug users has outpaced research. There is still a lack of studies centering on young Chinese drug users’ involvement in violent and non-violent criminal behaviors as well as the possible mental health-related concerns of drug use. We are curious about whether there are differences in terms of demographic characteristics and backgrounds, drug use histories, as well as mental health status between young Chinese drug users with a history of criminality, especially, violent ones, and those without similar background. We also wish to understand the experiences and stories of young drug users’ participation in criminal behaviors. To fill this notable gap, this study aims at extending the literature by exploring the criminal behaviors among young Chinese drug users.

## 2. Materials and Methods

A mixed methods research design was used in this exploratory and descriptive study, in order to achieve the abovementioned research goal. On the one hand, the quantitative analysis (Study 1) was designed to explore the overall situation of young Chinese drug users’ criminal behaviors. Study 1 attempted to find out the distinctive features of young drug users with criminalities, especially, with violent criminal behaviors, as compared with those without similar record. The qualitative analysis (Study 2), on the other hand, intended to investigate criminal experiences among young Chinese drug users comprehensively from the perspectives of these young drug users. Both of these studies were part of a larger research project implemented between 2013 and 2016, which was aimed at exploration into the Chinese drug users’ lives and drug use experiences. The project’s research protocol was approved by the School of Social and Behavioral Sciences at Nanjing University before the commencement of data collection. Administrators of the institutions in which the data were collected accepted their patients’ participation as protected by an approved Human Subjects Protocol Form.

### 2.1. Participants and Procedures

The sample of Study 1 were all patients in several Chinese compulsory residential drug treatment institutions. A stratification sampling approach was adopted, in order to identify respondents. First, we chose to do the survey in five provinces—two have the largest number of drug users in China; while another three, which are geographically connected to each other are on the eastern part, which is also the richest area in the country. Six male and three female compulsory drug treatment institutions were selected as the sites of investigation. Male compulsory drug treatment institutions are necessary more because men occupy a significantly higher proportion of drug users (any drug) in China [31]. In the two provinces with the largest number of drug use population, two male and two female institutions were first selected. Subsequently, another five institutions were chosen in the three eastern provinces, with four male and one female ones. In Chinese compulsory drug treatment institutions, patients are randomly assigned to a residential hall; one residential hall usually houses around 200 people. Therefore, we randomly chose two residential halls in each selected institution and asked all their residents to participate in the survey on a voluntary basis. Trained research assistants were on site to help participants seek for any clarification. All participants had 30 to 45 min to complete the questionnaire (including questions on the involvement of violent and non-violent crime, and history of drug use). We briefly introduced the purpose of the research to the participants and assured them of confidentiality prior to the completion of the questionnaire. They were allowed to leave the questionnaires blank without fear of being punished by the wardens. In total, 3473 questionnaires were distributed and 3239 were completed, with the participation rate of 93.3%. Among all respondents, 2300 (71.0%) were male while the rest 939 (29.0%) were female. The mean age of all respondents was 33.74 years. Since this specific study was only intended to investigate criminalities among young Chinese drug users, we selected respondents under the age of 30 to form the sample of Study 1. A total of 1347 young Chinese drug users were successfully recruited as respondents for the quantitative analysis.

The sample for Study 2 was also drawn from several compulsory drug treatment institutions in two provinces, one on the east coast, the richest part of China and the other on China’s southwest border which connects to the major opiate cultivation and production area—the Golden Triangle. We conducted three rounds of interviews for the larger research project. Administrative officers at the institutions offered to help identify and recruit interviewees. Adopting the criteria of maximum variation [32] which intends to include participants with multiple backgrounds, the researchers purposively recruited interviewees among the recommended candidates to maximize the diversity. The selected interviewees could afford to refuse to participate in the project; and eight declined to be interviewed. In total, 132 male and female drug users with various demographic backgrounds and distinct drug use histories completed the interviews. All interviewees voluntarily signed informed consent forms before participating in the interview. Again, according to the research goal, we only selected interviewees who: (1) were under the age of 30 and (2) reported criminal behaviors (both violent and non-violent) to be the participants of Study 2, which included five male and eight female drug users. With a relatively small sample, Study 2 did not aim at generalizing its findings, but just intended to provide an in-depth understanding of the story of how young Chinese drug users got involved in crimes. 

### 2.2. Measures

In accordance with the goals of this study, we asked several questions related to the participants’ criminal experiences and histories in the questionnaire of Study 1. First of all, we asked the following question, “How many times have you been charged, arrested, or formally prosecuted for the following issues?” We then listed several crimes (e.g., robbery, rape, drug-related crime, larceny, murder, physical injury) for participants to write the number of times they had committed in the past. If the participants had no criminal history, they could write “0” for all items. Those who reported criminal experiences were divided into two groups depending on whether they had committed violent crimes or not. We categorized physical injury, robbery, rape, and murder as violent crimes, while everything else was categorized as non-violent. We also asked a further question to determine whether participants had used drugs prior to engaging in criminal behaviors or vice versa. 

To assess the participants’ mental health, we adopted the Chinese version of Zung Self-Rating Depression Scale (SDS), which was originally developed by William W. K. Zung [33]. The SDS is a short and comprehensive self-administered instrument to measure the severity of respondents’ depression. It features 20 items, which rate the affective, physiological, and psychological symptoms associated with depression. Half of the items are positively worded and the other half are negatively worded. Each question is scored on a Likert-type scale ranging from 1 to 4 (“rarely”, “occasionally”, “often”, and “almost always”). The test scores, thus, range between 20 and 80. Multiplying this score by 1.25 gives a total score out of 100. Respondents’ depressed status can be clustered into four categories: the normal range (total score under 50), mild depression (total score between 50 and 59), moderate depression (total score between 60 and 69), and severe depression (total score of 70 and above) [34]. The higher the score, the more severe the depression experienced by the respondent.

In addition, we included several demographic and drug-use related items in the questionnaire, relating to gender, age, religious belief, marital status, level of education, drug initiation age, and crime type. We also asked two questions on whether the respondents had experienced emotional abuse and physical abuse in their childhood.

A semi-structured interview approach was adopted for data collection in Study 2. During the interviews, which were 60- to 90-min, audio-recorded, face-to-face, and in-depth, we asked several questions related to criminal experiences if the respondents had reported a criminal history. Interviewees were encouraged to describe their experiences in detail and to express their ideas and thoughts freely during the interview. Mandarin Chinese was used as the communication language. Five well-trained research assistants helped in the data collection. These five interviewers were trained before data collection and they also received ongoing supervision from the first author throughout the fieldwork.

### 2.3. Data Analysis

All data analyses of Study 1 were conducted using SPSS 22 (SPSS, Inc., Chicago, IL, USA). Bivariate analyses were conducted first to determine whether the young Chinese drug users’ criminal/non-criminal categories were associated with gender, age, religious belief, education level, marital status, history of emotional/physical abuse, age of drug initiation, as well as SDS continuous scores and categories. Next, those participants who reported criminal experiences were further divided into two categories based on crime type, namely whether they had committed violent crimes or not. We also conducted bivariate analyses on whether the two categories were associated with demographic background, mental health status, and drug use history. We used one-way analysis of variance (one-way ANOVA) and Scheffé’s post-hoc test for continuous variables and the Chi-square test for categorical variables. Binary logistic regression analyses were then used to identify possible factors influencing drug users’ participation in criminal behaviors, especially violent ones. In regression analyses, the continuous scores of SDS, rather than categories, were used when concerning the participants’ mental health influences on their criminalities. 

The qualitative data of Study 2 were analyzed through open coding and axial coding. The data were then reorganized into different categories and themes in line with our research goal. NVivo 10 software (QSR International Pty Ltd., Melbourne, Australia) was used during the data analysis process. Finally, the 13 participants’ narratives were interpreted and structured around the themes identified through the thematic analysis [35].

In data collection and analysis, the identities of participants were concealed. The questionnaires were self-completed anonymously, and quotes/descriptions of the interview participants were not collated to reconstruct the potential identity of any particular participants.

## 3. Results

### 3.1. Study 1

Among 1347 participants of Study 1, 333 (24.7%) reported that they had a history of criminal behavior. Table 1 shows the percentage of participants in criminal/non-criminal categories, divided by several factors related to demographics, drug use, and mental health. Chi-square analysis indicated that there was significant gender difference between the two categories (χ^2^ = 40.93; *p* < 0.001). The participants with a history of criminality were largely male. The two categories did not differ in average age, being around 25 years old in both categories. Chi-square analysis also yielded significant difference for religious beliefs across criminal/non-criminal categories (χ^2^ = 10.48; *p* < 0.01). More participants with criminalities reported to hold religious beliefs. More than half of participants had completed nine years of education (middle school) or less, and there was no significant difference between the two categories in terms of education. Chi-square analysis found a significant difference in marital status between the two categories (χ^2^ = 2.17; *p* < 0.1). People who had a criminal history were more likely to be married. Drug users who had involved in crimes reported significantly higher levels of suffering from emotional abuse (χ^2^ = 22.34; *p* < 0.001) and physical abuse (χ^2^ = 19.82; *p* < 0.001) in childhood. In addition, one-way ANOVA showed drug initiation at a significantly younger age among drug users with a criminal history (F = 13.39; *p* < 0.001). However, there was no significant difference in SDS scores as well as categories between drug users with and without a history of criminality. Most respondents reported no or mild depression. Very few participants expressed that they were severely depressed, which indicated that depression was not a serious mental health problem among these young Chinese drug users, regardless of whether they had a criminal history.

A binary logistic regression analysis was conducted to test the factors influencing the likelihood of drug users’ involvement in criminal behaviors (see Model 1 in Table 2). The results show that male drug users with older ages, religious beliefs, and lower education levels were more likely to commit crimes. Additionally, those who had experienced emotional or physical abuse were also found to be more likely to engage in criminal behaviors. The younger the age of drug initiation, the greater the likelihood of their involvement in crime. Furthermore, drug users who experienced more severe levels of depression (e.g., higher SDS scores) were also more likely to commit crimes.

Of the 333 participants with a history of criminality, 165 (49.5%) had committed violent crimes, including physical injury, robbery, rape, and murder, while the rest 168 (50.5%) reported only participating in non-violent criminal behaviors, which included drug-related crimes (e.g., drug dealing, drug trafficking, sheltering drug use, and drug possession), sex crimes besides rape, larceny, possession of an illegal weapon, forgery, arson, and misbehavior during probation or parole. Those who reported both violent and non-violent criminal behaviors were categorized into the violent group. Table 3 presents a comparison across groups of drug users with violent/non-violent criminal behaviors, in terms of their varying demographics, mental health status, and drug use histories. Chi-square analysis showed that the gender difference between the two groups was significant (χ^2^ = 41.50; *p* < 0.001), highlighting that male users were more likely to commit violent crimes. However, there were no significant differences between the two groups regarding age, religious belief, education level, or marital status. Drug users with a non-violent criminal history presented emotional abuse experiences more often than those with violent criminal backgrounds (χ^2^ = 5.69; *p* < 0.05); while there was no significant difference between the groups in terms of physical abuse experiences. Participants’ average age at drug initiation was around 19 and there was no significant difference between the two groups. Chi-square analysis indicated significant difference in terms of whether participants initiated drug use prior to criminal behaviors or vice versa (χ^2^ = 19.27; *p* < 0.001). Drug users with a history of violence were more likely to have committed crimes prior to drug initiation. Participants from the violent group committed both violent and non-violent crimes. Physical injury was the most commonly mentioned (83.6%). In addition, 26.7% violent group participants reported that they had a drug-related criminal history. Non-violent group participants committed only non-violent crimes, among which, drug-related crimes were reported by 75.0% participants. There was no significant difference between the violent and non-violent groups regarding SDS scores and categories. 

We also conducted a binary logistic regression analysis to determine the factors influencing the likelihood of drug users committing violent/non-violent criminal behaviors (see Model 2 in Table 2). The results showed that, comparatively, older male drug users with religious beliefs were more likely to commit violent crimes. Interestingly, drug users who had not experienced emotional abuse were found to be more likely to be involved in violent crimes. In addition, those who initiated drug use at a younger age had greater likelihood to engage in violent crimes. The drug users who committed crimes prior to drug initiation were more likely to commit violent crimes. Regarding mental health status, those who experienced more severe depression levels, namely higher SDS scores, tend to commit more violent crimes.

### 3.2. Study 2

The participants were five male and eight female drug users aged 30 or under who had reported criminal behaviors. The demographic and drug use-related features of the 13 respondents are listed in Table 4. Their age ranged from 16 to 30, with an average of 23.6 years. All participants but one had only completed middle school education or less. Only one participant was married. They generally initiated drug use at a young age: ten had started at 20 years of age or younger and the average initiation age was 18.6 years. All of them had committed non-violent crimes, while six also had committed violent crimes.

#### 3.2.1. Involvement in Criminal Behavior

Three of the five male participants were members of youth gangs and had started their criminal career before the age of 20. They all described their involvement in several types of criminal behavior, both violent and non-violent. Their lives centered on criminalities, among which, physical injury through public flights and robbery were the most commonly cited experiences. In addition to violent behaviors, these gang members also had participated in several non-violent criminal behaviors, e.g., drug trafficking and dealing, possession of weapons, usurious loans, gambling, etc. One of them stated that:

*I was following a “boss” at that time. That was in my early twenties. We had several people who worked for the “boss”. We ran lots of businesses including drug trafficking and dealing, gambling, a usurious loan business, etc. We also provided paid “protection” for Karaoke rooms and night clubs. Sometimes, we had to fight against other gangs, to protect our places and markets. Of course, there were injuries. We had weapons, like guns, but we seldom used them actually; they were only for deterrence. (Jiang (* *“Jiang” is the pseudonym to replace the real name of the participant, in order to protect his privacy. The same hereinafter), male, 30 years old, with five-year history of methamphetamine use).*

Each of the other two male participants reported involvement in affray and robbery, although they were not gang members. One mentioned that he had been imprisoned for robbery:

*I was sentenced for two years of imprisonment for robbery. I did it with a few friends. We actually did not mean to fight the victim, it was just for money. We needed money to live and to buy drugs. (Huo, male, 30 years old, with nine-year history of heroin use, and occasional methamphetamine use).*

Only one out of eight female participants reported experience of violence. Similar to her male counterparts, she had also belonged to a youth gang. She said that she had committed affray, which involved weapon possession and caused physical injury:

*It was when I was with my friends. As a group, we fought against another group. Both sides had guns. I really had no idea of where each group got the gun from. The guys handled the weapons as for us girls just joined them. (Tong, female, 25 years old, with one-year history of methamphetamine use).*

The other seven female drug users only had committed non-violent crimes. Three had been drug traffickers and dealers. These female drug dealers all emphasized that they only were involved in small-scale trafficking and dealing, to support their own drug use. They denied involvement in large-scale dealing, as this was considered a “men’s job”. One of them reported:

*I have used drug for many years. I need it every day, so I need money. Drug users mainly do drug dealing and trafficking to earn money, because it is a quick fix and needs no specialized skills. It is an easy way to make money. I, sometimes, do it just to support my own drug use. I don’t consider myself to be a heavy-duty drug trafficker. I think capable men can do that kind of job and not women like me. (Su, female, 25 years old, with eight-year history of poly drug use, mainly methamphetamine and cocaine, and occasional use of ketamine).*

Apart from drug dealing and trafficking, one female participant, Wan aged 22 years old with three-year history of methamphetamine use, also reported that she received a half-year sentence for “sheltering other people to use drugs”.

Other three female participants reported larceny. One mentioned stealing mobile phones together with her boyfriends:

*We need the money to buy drugs, that’s why we, sometimes, steal. I remember one day my boyfriend and I, together with other friends, went to a cybercafé and stole several mobile phones. We then sold them and got money. (Dan, female, 21 years old, with five-year history of heroin and methamphetamine use).*

Besides the one gang member, three drug traffickers/dealers, and three larcenists, the rest female drug user reported involvement in illegal gambling as well as sex industry, which Chuan aged 20 years old with one-year history of methamphetamine use described as “providing places for some friends to engage in prostitution.”

#### 3.2.2. Drug Use, Mental Health Problems, and Criminal Career

The three male and one female drug users who admitted to being members of a youth gang very much considered drug use to be part of their criminal career. They were involved in several deviant and criminal behaviors, and drug use as narrated by one of them:

*We did many things, including extorting money, running sex work and gambling, usurious loans, trafficking and dealing drugs, and gathering crowds in order to engage in affray. I had several subordinates in the gang. Wherever I went, my boys treated me with drugs and ladies. That was just my lifestyle. I like drugs, they make me happy. (Ling, male, 23 years old, with three-year history of methamphetamine use).*

One of the four members of the gang pointed out that drugs, especially methamphetamine or other types of non-opiate synthetic drugs, helped them to “feel powerful and energetic”, thus increasing the likelihood of engaging in criminal behaviors. He asserted as thus:

*I think it (methamphetamine) has some psychological effects. After using it, I could do anything that I wouldn’t dare do before taking it. I would feel powerful, and could afford to do anything. I fought people after using methamphetamine. I feared nothing. That feeling was good and a sense of achievement so to say. (Xia, male, 24 years old, with six-year history of poly drug use, mainly methamphetamine and cocaine, also occasional use of other non-opiates).*

Interestingly, all four gang members reported that they started their criminal career with delinquency and minor offenses, such as shoplifting and prostitution. This narration is attributed to Tong (*female, 25 years old, with one-year history of methamphetamine use*) while Jiang (*male, 30 years old, with five-year history of methamphetamine use*) hinted on their involvement in illegal gambling all as initial behaviors prior to their initiation into drug use. These findings also demonstrate that drug use was seen as an aspect of their criminal lifestyle.

Another two male participants who reported robbery and affray experiences held that their criminal behaviors had nothing to do with their drug use. They regarded them as two separate things: “No, robbery was robbery. I don’t think it had a relationship with my methamphetamine use”, said one of the two men (*Yang, male, 21 years old, with six-year history of methamphetamine use*). They mentioned that they had been involved in criminal behaviors before their use of drugs began. They believed that both drug use and criminal behaviors were part of their lives, like all other youth gang members.

The other seven female participants with non-violent criminal experiences, such as drug dealing or larceny, all recognized that their criminal behaviors were due to their drug use. They generally admitted that they committed crimes as a consequence of drug use, as they had to make money to support their drug use.

## 4. Discussion

The developmental periods of adolescence and young adulthood are characterized by heightened vulnerability to risk-taking behaviors, including both drug use and criminal behavior [1,2,3,4,36,37]. The results from Studies 1 and 2 both show the close relationship between drug use and crime among young people. Results from Study 1 showed that, compared to young Chinese drug users who had no criminal history, those with criminalities were more likely to be men, married, with religious belief, with both physical and psychological abused experiences, and having younger drug initiation age. However, within the young drug using population with criminalities, compared to the ones who only had committed non-violent crimes, those who had committed violence were primarily men, with no history of emotional abuse, and committing crimes prior to their initiation in drug use. Logistic regression analyses showed that older male drug users who had religious beliefs and initiated drug at younger age were the most likely to be involved in both violent and non-violent criminal behaviors. Drug use, in one way or another, may lead to considerable mental disorders [13,14,15,16]. Among which, depression is the most commonly found, and previous studies demonstrate a close relationship between depression and drug use [38,39,40,41,42,43,44]. In the current study, while the results did not show a very severe level of depression among young Chinese drug users, young drug users’ mental health status was found to have a significant influence on their violent and non-violent criminal behaviors. Those with more severe depression were more likely to commit crimes, especially violent ones. This finding is consistent with that of previous studies which suggest that both drug use and mental health disorders are associated with criminal behaviors [17,18,19,45,46]. Results from Study 2 illustrated that men were more likely to join youth gang and committed violent crimes, and explained their drug use as a part of criminal career; while women committed drug related crimes such as drug dealing and trafficking, or larceny in order to obtain money to feed their drug habit. This finding is also consistent with several previous empirical studies. Men are more likely than women to join criminal gangs [47] and also committed more violent and serious crimes than women [48,49].

The current study has several theoretical and practical implications. First, the finding of the current study is consistent with developmental theories which argue that young people’s early, minor delinquent acts and anti-social behaviors are predictors of subsequent substance abuse initiation and dependence, as well as substance-related clinical disorders [30,50,51,52,53]. For instance, Study 1 shows that among the drug users who had a criminal history, those who had committed crimes prior to their initiation in drug had a greater likelihood of committing violent crimes. Study 2 further supports this observation. The young people who reported a history of violence were mostly those who started with delinquency or minor offenses, joined a gang or started a criminal career, and then started taking drugs. The initiation and increasing use of drugs predicted subsequent violent and non-violent behaviors [54]. Therefore, these young people were growing up with their drug and their criminal careers intertwined together. Similar to what has been found through Study 1, prior research reported that violent and non-violent offenses are seen by these chronic offenders as elements of a larger syndrome of delinquent and antisocial behavior which includes not only several types of crimes, but also substance use, reckless driving, bullying, and sexual promiscuity [55]. 

“Victim to offender cycle” which is proposed as an explanation of offending is regarded as the second theoretical concern [56,57,58,59]. According to the findings of Study 1, drug users with a history of physical and/or emotional abuse are more likely to commit crimes. There is evidence that childhood maltreatment drives individuals to take part in delinquency or later criminal activities [60,61,62,63,64]. A considerable number of theories explain that experiencing violence in childhood leads to involvement in crime and delinquency later in life [60,64]. One of the most cited explanations comes from the social learning theory, which argues the abused children are more likely to learn to be aggressive from the abusers through imitating, and then pass on what they have learned to others after they grow up [64,65]. However, in terms of crime type, Study 1 found that experience of physical abuse had no effect on whether the respondents committed violent or non-violent crimes; while those who had experienced emotional abuse were more likely to commit non-violent crimes. In this respect, these findings indicate that not all adverse childhood experiences are a predictor of drug users’ involvement in violent behaviors.

The intervention implication comes from the concern of close link among drug use, negative mental health outcomes, and criminal behavior. Since drug use can lead to serious mental health problems [13,14,15,16], it has been suggested that integrated mental health and substance use services be launched to help the drug user population [20]. Early intervention programs, which are much needed in China, can help drug users deal with various mental health issues such as depression and social withdrawal [14,15,16]. In addition, both Studies 1 and 2 show that drug users who incurred mental health problems are more likely to commit both violent and non-violent crimes. That is, especially, the case going by the findings from Study 2, which prove that drug use can cause mental health disorders, thus increasing the likelihood of criminal activity. Therefore, comprehensive intervention programs to drug users not only can solve their mental health problems as well as drug dependence, but also are ways to reduce or prevent young Chinese drug users’ involvement in criminal behaviors. 

This study is not without limitations. First, as a descriptive analysis, while Study 1 only indicates that drug use and crime are correlated positively, this association does not necessarily imply a simple causal relationship. Instead of using a cross-sectional design, a prospective follow-up or panel study design is recommended to understand different pathways into drug use and crime among young people. Second, the sample of Study 2 is relatively small, and that the qualitative analysis can only provide a glimpse of the experience of a selected group of young Chinese drug users.

## 5. Conclusions

To conclude, drug use, a critical public health concern, is found through this exploratory and descriptive study that has a possible impact on violent and non-violent criminal behaviors among young Chinese drug users. A mixed method design not only helps grasp a better understanding of distinctive features of young Chinese drug users with criminal behaviors, especially, with violence; but also reports vivid stories of involvement of violent or non-violent crime among these drug users. As discussed, the findings of the current study are consistent with developmental theories and the “victim to offender cycle.” To break the cycle of drugs and crime afflicting so many of young people, a more effective early intervention or remedial program that addresses complex and multiple needs should be sought by mental health and youth justice practitioners and policymakers. Given the fact that the current study gives a descriptive account of the link between drug use and violent or non-violent behaviors among young people, further research, in China, is needed to understand more about the drug-crime/violence cycle by using a more rigorous research design and larger sample population. 

## Figures and Tables

**Table 1:** Comparison across Drug Users with/without a History of Criminal Behaviors (*n* = 1347).

Variable		Criminal Behavior *n* = 333 *n* (%)/M (SD)	No Criminal Behavior *n* = 1014 *n* (%)/M (SD)
Gender ***	Female	68 (20.4%)	401 (39.5%)
	Male	265 (79.6%)	609 (60.1%)
	N/A	0	4 (0.4%)
Age		25.92 (3.035)	25.83 (3.258)
Religious belief **	No	263 (79.0%)	856 (84.4%)
	Yes	52 (15.6%)	93 (9.2%)
	N/A	18 (5.4%)	65 (6.4%)
Education level	Primary school and under	76 (22.8%)	204 (20.2%)
	Middle school	148 (44.4%)	460 (45.4%)
	High school (including vocational high school)	75 (22.5%)	236 (23.3%)
	Junior college and above	29 (8.7%)	99 (9.9%)
	N/A	5 (1.5%)	15 (1.5%)
Marital status ^+^	Single (including divorced and widowed)	198 (59.5%)	637 (62.8%)
	Married (including cohabitation)	134 (40.2%)	356 (35.1%)
	N/A	1 (0.3%)	21 (2.1%)
Emotional abuse ***	No	226 (67.9%)	793 (78.2%)
	Yes	103 (30.9%)	184 (18.1%)
	N/A	4 (1.2%)	37 (3.6%)
Physical abuse ***	No	257 (77.2%)	858 (84.6%)
	Yes	72 (21.6%)	116 (11.4%)
	N/A	4 (1.2%)	40 (3.9%)
Drug initiation age ***		19.5 (3.165)	20.31 (3.554)
SDS		48.4481 (10.92)	46.8206 (10.69)
	Normal range (under 50)	147 (44.1%)	486 (47.9%)
	Mildly depressed (50–59)	87 (26.1%)	216 (21.3%)
	Moderately depressed (60–69)	28 (8.4%)	83 (8.2%)
	Severely depressed (70 and over)	8 (2.4%)	12 (1.2%)
	N/A	63 (18.9%)	217 (21.4%)

^+^ *p* < 0.1, ** *p* < 0.01, *** *p* < 0.001.

**Table 2:** Results of Binary Logistic Regression Analyses.

Variable	Model 1 *n* = 1347 (Criminal = 1)	Model 2 *n* = 333 (Violent = 1)
Gender (Male = 1)	0.223 ***	0.344 ***
Age	0.079 *	0.108 ^+^
Religious belief (Yes = 1)	0.080 **	0.100 ^+^
Education level	−0.083 **	0.049
Marital status (Married = 1)	0.032	−0.017
Emotional abuse (Yes = 1)	0.097 **	−0.149 *
Physical abuse (Yes= 1)	0.099 **	−0.017
Drug initiation age	−0.162 ***	−0.186 **
Crime first or drug use first (Crime first = 1)		0.296 ***
SDS	0.053 ^+^	0.140 *

^+^ *p* < 0.1, * *p* < 0.05, ** *p* < 0.01, *** *p* < 0.001.

**Table 3:** Comparison across Drug Users with a History of Violent/Non-Violent Criminal Behaviors (*n* = 333).

Variable		Drug Users with Violent Criminal History *n* = 165 *n* (%)/M (SD)	Drug Users with Non-Violent Criminal History *n* = 168 *n* (%)/M (SD)
Gender ***	Female	10 (6.1%)	58 (34.5%)
	Male	155 (93.9%)	110 (65.5%)
Age		26.33 (3.00)	25.51 (3.02)
Religious belief	No	132 (80%)	131 (78%)
	Yes	27 (16.4%)	25 (14.9%)
	N/A	6 (3.6%)	12 (7.1%)
Education level	Primary school and under	35 (21.2%)	41 (24.4%)
	Middle school	80 (48.5%)	68 (40.5%)
	High school (including vocational high school)	37 (22.4%)	38 (22.6%)
	Junior college and above	11 (6.6%)	18 (10.7%)
	N/A	2 (1.2%)	3 (1.8%)
Marital status	Single (including divorced and widowed)	97 (58.8%)	101 (60.1%)
	Married (including cohabitation)	68 (41.2%)	66 (39.3%)
	N/A	0	1 (0.6%)
Emotional abuse *	No	122 (73.9%)	104 (61.9%)
	Yes	41 (24.8%)	62 (36.9%)
	N/A	2 (1.2%)	2 (1.2%)
Physical abuse	No	130 (78.8%)	127 (75.6%)
	Yes	33 (20.0%)	39 (23.2%)
	N/A	2 (1.2%)	2 (1.2%)
Drug initiation age		19.56 (3.087)	19.45 (3.248)
Crime first or drug use first ***	Drug use first	96 (58.2%)	135 (80.4%)
	Crime first	69 (41.8%)	33 (19.6%)
Crime Types (Violent)	Physical injury	138 (83.6%)	0
	Robbery	31 (18.8%)	0
	Rape	9 (5.5%)	0
	Murder	6 (3.6%)	0
Crime Types (Non-violent)	Drug-related	44 (26.7%)	126 (75.0%)
	Sex-related (other than rape)	2 (1.2%)	6 (3.6%)
	Larceny	29 (17.6%)	23 (13.7%)
	Illegal weapon possession	18 (10.9%)	12 (7.1%)
	Forgery	4 (2.4%)	1 (0.6%)
	Arson	4 (2.4%)	1 (0.6%)
	Misbehavior during probation or parole	18 (10.9%)	20 (11.9%)
SDS		49.540 (11.22)	47.3556 (10.55)
	Normal range (under 50)	68 (41.2%)	79 (47%)
	Mildly depressed (50–59)	50 (30.3%)	37 (22%)
	Moderately depressed (60–69)	11 (6.7%)	17 (10.1%)
	Severely depressed (70 and over)	6 (3.6%)	2 (1.2%)
	N/A	30 (18.2%)	33 (19.6%)

* *p* < 0.05, *** *p* < 0.001.

**Table 4:** Key Features of Participants in Study 2 (*n* = 13).

Variable		*n* = 13 *n* (%)/M (Range)
Gender	Female	8 (61.5%)
	Male	5 (38.5%)
Age		23.6 (16–30)
Education level	Primary school and under	2 (15.4%)
	Middle school	10 (76.9%)
	High school (including vocational high school)	0 (0%)
	Junior college and above	1 (7.7%)
Marital status	Single (including divorced and widowed)	12 (92.3%)
	Married (including cohabitation)	1 (7.7%)
Drug initiation age		18.6 (14–24)
Drug used	Heroin	4 (30.8%)
	Methamphetamine	13 (100%)
	Other	3 (23.1%)
Criminal Experiences	Violent crime	6 (46.2%)
	Non-violent crime	13 (100%)
